# The Effects of Cognition and Vision While Walking in Younger and Older Adults

**DOI:** 10.3390/s24237789

**Published:** 2024-12-05

**Authors:** Trong Pham, Meagan Suen, Young-Hee Cho, Vennila Krishnan

**Affiliations:** 1 Office of Research and Economic Development, California State University Long Beach, Long Beach, CA 90840, USA; trong.pham@csulb.edu; 2 Department of Psychology, California State University Long Beach, Long Beach, CA 90840, USA; msuen@mednet.ucla.edu (M.S.); young-hee.cho@csulb.edu (Y.-H.C.); 3 Department of Physical Therapy, California State University Long Beach, Long Beach, CA 90840, USA

**Keywords:** dual-task walking cost, vision, cognitive–motor interference, Stroop, serial-subtraction, older adults

## Abstract

This study investigated how various cognitive tasks and visual challenges affect dual-task walking costs (DTWC) in younger and older adults. Twenty younger adults (Mean_age_ = 22.25, SD = 3.04, 4 males) and eighteen older adults (Mean_age_ = 71.75, SD = 5.17, 7 males) completed single-task walking and dual-task walking. The dual tasks involved walking while performing either (a) serial-subtraction by 3s or (b) a Stroop task. Both single tasks and dual tasks were performed under both normal vision and peripheral-vision-loss conditions. Results showed no significant three-way interaction but two significant two-way interactions: DTWC for step-length was greater during Stroop compared to serial-subtraction, (a) more in older adults regardless of vision (*p* = 0.022) and (b) more under peripheral-vision-loss regardless of age (*p* = 0.033). In addition, DTWC for various gait parameters was greater under (a) Stroop compared to serial-subtraction, (b) peripheral-vision-loss compared to normal vision, and (c) older adults compared to younger adults. These findings suggest that, when engaging in a cognitively demanding task, older adults place greater emphasis on maintaining gait compared to younger adults, likely to offset the negative impacts of additional cognitive load and deteriorated vision. Future research should further examine how different cognitive tasks and visual challenges interact across age groups.

## 1. Introduction

The suboptimal performance in walking while simultaneously performing another task compared to walking only is referred to as a dual-task walking cost (DTWC) [1]. DTWC occurs because attention must be divided, leading to cognitive–motor interference (CMI) [2]. Studies have shown that types of cognitive task [3] and impaired vision [4] influence gait. Although the impact of impaired vision on gait has been studied extensively, only a few studies have investigated its impact on gait while performing another task in both older and younger adults [5,6].

Past studies have demonstrated the impact of poor cognitive function on gait [7,8]. Executive function (EF) is a critical cognitive factor influencing gait performance [9] and refers to higher-order cognitive skills used to direct goal-oriented actions [10]. The development of EF has been linked to the structural integrity and activity of the frontal lobes [7]. Past studies have found decline in EF among older adults is associated with slower gait speed, increased stride variability, poorer performance on complex mobility tasks, and increased fall risk [11,12,13]. A possible explanation for decline in EF among older adults is the age-related shrinkage of the prefrontal cortex, which affects higher-level cognitive function [2] and consequently, reduces the cognitive resources allocated to gait.

One sub-component of EF strongly affected by aging is inhibition [14], which refers to the ability to tune out irrelevant stimuli [15], as demonstrated in tasks such as the Stroop (STR) task (i.e., reciting the color of the ink in which a color-word is printed) [16]. Deficits in response-inhibition can lead to failure to provide sufficient attention to gait in complex environments with distractions [2]. Another component of EF impacted by aging is working memory, which is a limited-capacity system that actively maintains task-relevant information for a brief period [17,18]. An example of working memory in action could be attempting to calculate the cost of all the items in a basket at the supermarket. Previous studies have found that the processing components of working memory (e.g., mental operations) as opposed to the maintenance components responsible for storage are more greatly impacted by age [19,20]. Thus, deficits in working memory can lead to decreased ability in walking, but not as much as deficits in inhibition.

The effect of age-related changes in cognition on gait is accentuated under DT conditions [1]. Previous studies have explored how different cognitive tasks impact gait performance during DT [3,21]. It was found that the Stroop task, a measure of inhibitory control, resulted in greater DTWC in gait velocity in younger adults compared to other types of cognitive tasks (e.g., working memory task) [21]. However, the effects of different types of cognitive tasks on multiple gait parameters other than gait velocity have not been fully explored [1]. Gait is inherently multidimensional and characterized by interdependent spatial and temporal features. While gait velocity is more commonly explored, other metrics, such as double-limb support, step length, and cadence, are essential for capturing the complexity of gait and can highlight subtle imbalances or efficiency differences even in adults without impairments [22,23]. Additionally, experimental results of cognitive–motor dual-tasking are often ambiguous, particularly when distinct age groups are compared [24,25]. Specifically, while it remains undisputed that walking speed decreases under DT conditions compared to single-task, some studies have found decreases to be more pronounced in older adults [26,27], while others have demonstrated no differences between younger and older participants [28,29]. Therefore, it is important to investigate the impact of different cognitive tasks on multiple gait parameters under the DT paradigm and compare the results of older adults to those of younger adults in order to better understand the effects of age-related change in cognition on older adults’ gait performance.

Another factor impacting gait is impaired vision [4,30]. Given that individuals typically direct their gaze and attention toward the task at hand, this implies a close interconnection between vision, attention, and body movement [31,32]. Studies have shown that blurred vision and peripheral-vision-loss (PVL) can deteriorate postural control [33] and gait [34,35]. Blurred vision increases the risk for loss of balance during walking due to diminished visual acuity and depth perception [33,34], while PVL disrupts spatial awareness during walking [36]. In one study, PVL caused participants to deviate from their intended path while walking [35]. Like cognition and gait, vision is also affected by age-related changes, thus potentially exacerbating DT performance in older adults. Despite the evidence supporting the adverse impact of impaired vision on gait, only a few studies have explored its effects on DTWC among older adults [6,37,38].

The purpose of this study is to examine the effects of domain-specific cognitive tasks, various visual conditions, and age on various walking parameters. Unlike most previous literature, which focused solely on the DT cost of either gait velocity [21] or postural stability [37], this study examines the DT cost across multiple gait parameters, such as step length, double support, velocity, and cadence. We hypothesized that older adults, compared to younger adults, would exhibit a greater DTWC in gait while performing an inhibitory task rather than a working memory task, and this greater DTWC in gait would be exacerbated with PVL.

## 2. Materials and Methods

### 2.1. Participants

Twenty younger adults and 22 older adults were recruited for this study. Younger adults were students at California State University, Long Beach (CSULB), and older adults were recruited from the Long Beach Senior Center. Inclusion criteria were as follows: (a) be between the ages of 18 to 30 for younger adults and over the age of 60 for older adults, (b) be community-dwelling, (c) be able to walk independently, (d) have normal or corrected-to-normal hearing and vision, (e) be free of cognitive impairment, (f) have no history of neurological disorders, and (g) be free of any orthopedic or neurologic conditions which could affect gait. Four older adults due to cognitive impairment assessed by Mini-Cog (see below) were excluded, resulting in 20 younger adults (Mean_age_ = 22.25, SD = 3.08, 4 males) and 18 older adults (Mean_age_ = 71.75, SD = 5.17, 7 males). This study was approved by the Institutional Review Board (IRB) at CSULB and was conducted according to the Declaration of Helsinki [39].

### 2.2. Measures

Mini-Cognitive Assessment. Older adults completed the Mini-Cognitive (Mini-Cog) Assessment as a screening tool to detect possible cognitive impairment [40]. The Mini-Cog assesses domains such as memory, language comprehension, visual-motor skills, and EF [40]. The Mini-Cog is a quick cognitive screening tool that has been shown to have 67% sensitivity and 83% specificity when detecting cognitive impairment among older adults [40]. The assessment comprises two tasks: a clock drawing task and a 3-word recall task. A total score of 0–2 indicates a higher likelihood of cognitive impairment, while a score of 3–5 indicates a lower likelihood of cognitive impairment.

Gait. The ZenoMat ‘Platinum’ 16 ft. electronic walkway was used to assess gait. The mat is 12 by 2 ft. (3.66 × 0.61 m) and contains 13,824 force sensors capable of recording walking data from the first to last footstep across the length of the mat. Data from the ZenoMat that are normalized using anthropometric data of height and leg length collected at the start of data collection were analyzed using the Protokinetics Movement Analysis Software (PKMAS-ProtoKinetics, Havertown, PA, USA).

Cognition. A serial-subtraction (SS) task was used to assess working memory and information processing. Participants were given a 3-digit number between 100 and 200 and instructed to count backwards by intervals of 3s. Different 3-digit numbers were used in each SS by 3s trials to reduce the possibility of practice effects. The Stroop (STR) tsk was used to assess inhibitory control. Participants were given a sheet with eight rows and five columns of incongruent color-word pairings and were instructed to name the ink color in which the word was printed. To avoid potential practice effects, participants were presented with two versions of the STR task: one for single tasks and one for dual tasks. Both versions contained the same number of colors—red, blue, green, black, brown, purple, and yellow—but in random order.

Vision. During trials, all participants wore laboratory goggles. For normal vision (NV) conditions, they wore a pair of clear goggles. For PVL conditions, they wore goggles which had been occluded with black tape to eliminate peripheral vision [38,41,42]. If the participants wore prescription glasses, they were instructed to wear goggles over their glasses.

### 2.3. Procedures

Single Task Procedures: Single-task trials consisted of a walking task, STR task, and SS by 3s. Each task was performed twice: once under the NV condition and once under the PVL condition. The order in which participants perform these tasks was counterbalanced. For the walking task, participants were asked to walk at comfortable speed across the ZenoMat, turn around, and then walk back to the starting point. The ZenoMat was connected to a laptop running the PKMAS program, which recorded the participant’s footsteps and used these data to calculate gait scores based on parameters such as step length, double support, velocity, and cadence. For the STR task, participants were instructed to sit on a chair and list the ink-color of each incongruent color-word pairing for 20 s. Participants were instructed to hold the Stroop chart at a comfortable reading distance. For SS by 3s, participants were also instructed to sit on a chair and count backward by 3s starting from a predetermined 3-digit number for 20 s.

Dual-Task Procedures: Upon completion of all single tasks, participants took a 10-min break and then performed dual-task trials. The order in which participants completed dual-tasks was counterbalanced. For DT trials, participants performed the walking task and each cognitive task simultaneously (walking + STR, walking + SS by 3s) under NV and PVL conditions (refer to [37] for more detailed steps regarding procedures). For walking + STR, participants were instructed to walk at a comfortable pace across the ZenoMat and back to the starting point while simultaneously performing a different version of the STR task. For walking + SS, participants were also instructed to walk across the ZenoMat and back while simultaneously performing the SS task using a different 3-digit starting number. Gait scores were computed through the same process as the walking-only trial.

### 2.4. Analysis

An a priori analysis using MorePower 6.0 indicated that a minimum sample size of 18 was needed for each participant group to obtain statistical power at the recommended 0.80 level and an alpha value of 0.05 and an effect size of 0.5. Shapiro–Wilk tests showed that data were normally distributed, *p* > 0.05 [43].

DTWC [21,44] was calculated using the following formula:

DTWC (%) = (single task performance-dual task performance)/(single task performance) × 100%.

A separate mixed 3-way ANOVA was conducted to determine the impact of cognitive task (SS vs. STR), vision (normal vision vs. PVL), and age (younger vs. older) on each gait parameter of step length, cadence, gait velocity, and double support. Cognitive tasks and vision conditions were within-subject variables and age was a between-subject variable. Significant results were followed by follow-up analyses, and Benjamin–Hochberg correction [45] was used to control for Type I error. Analyses were conducted using SPSS version 29 for Windows (SPSS Inc., Chicago, IL, USA).

## 3. Results

**Figure 1:**
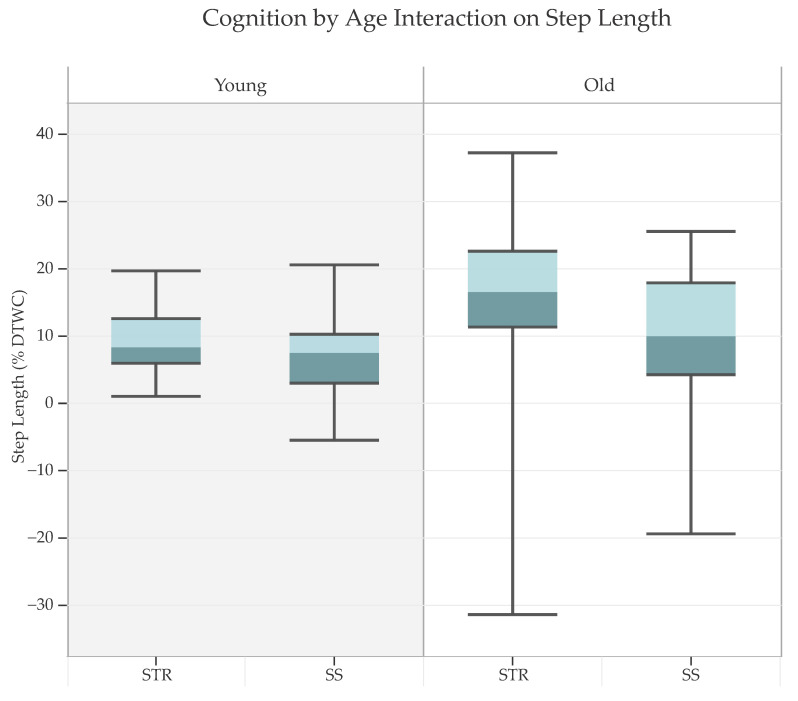
*Two-way Interaction effect of cognition and age on dual-task walking cost (DTWC) for step length* (*p* = 0.021), where performing the STR task resulted in a greater reduction in step length compared to the SS task, with this effect being more pronounced in older adults. Note: Boxes represent interquartile ranges, the lighter shade of color represents upper 50% of the data, and error bars represent minimum and maximum values. Larger positive *y*-axis value indicates greater DTWC, reflecting reductions in step length. STR = Stroop; SS = serial subtraction.

The purpose of this study was to investigate a three-way interaction among cognitive task, vision, and age on various gait parameters. Table 1 presents means and standard deviations of various gait parameters and DTWCs for different cognitive tasks and vision conditions for younger and older adults. The results of the mixed ANOVA showed no significant three-way interaction for all the gait parameters: step length [F(1, 36) = 0.76, *p* = 0.391, partial η^2^ = 0.02], double support [F(1, 36) = 0.58, *p* = 0.450, partial η^2^ = 0.02], velocity [F(1, 36) = 0.70, *p* = 0.409, partial η^2^ = 0.02], and cadence [F(1, 36) = 0.01, *p* = 0.944, partial η^2^ < 0.001]. However, a significant two-way interaction of cognitive task (STR vs. SS) by age (younger vs. older) was found only for step length, F(1, 36) = 5.80, *p* = 0.021, partial η^2^ = 0.14 (Figure 1). Follow-up analysis on this two-way interaction revealed greater DTWC step length (i.e., reduced step length) when performing STR than SS for both older adults, t(17) = −4.25, *p* < 0.001, CI [−8.91, −3.00], and younger adults, t(19) = −4.08, *p* < 0.001, CI [−3.67, −1.18], indicating that performing the STR task led to a greater reduction in step length than performing the SS task, with this effect being more pronounced in older adults.

**Figure 2:**
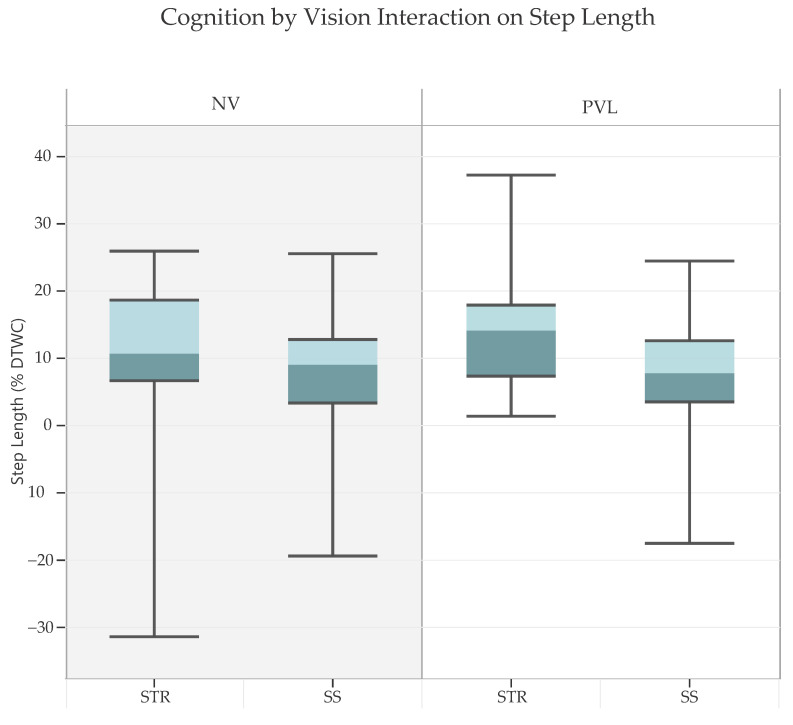
*Interaction effect of cognition and vision on dual-task walking cost (DTWC) for step length* (*p* = 0.033) demonstrated that the STR led to a greater reduction in step length than the SS, particularly when peripheral vision was obstructed. Note: Positive *y*-axis values indicate greater DTWC, reflecting reductions in step length. Boxes represent interquartile ranges, the lighter shade of color represents upper 50% of the data, and error bars represent minimum and maximum values. STR = Stroop; SS = serial subtraction; NV = normal vision; PVL = peripheral vision loss.

There was also a significant two-way interaction between cognitive task (STR vs. SS) and vision (NV vs. PVL) only for step length, F(1, 36) = 4.92, *p* = 0.033, partial η^2^ = 0.05 (Figure 2). Follow-up analysis showed that DTWC for step lengths were greater when performing STR than SS under both normal vision, t(37) = −2.88, *p* = 0.007, CI [−4.57, −0.79], and obstructed peripheral vision, t(37) = −5.062, *p* < 0.001, CI [−7.72, −3.31]. However, this effect was more pronounced under PVL conditions, indicating that STR led to greater reduction in step length compared to SS, especially when peripheral vision was obstructed. The two-way interaction between vision and age was not significant for any of the gait parameters.

**Figure 3:**
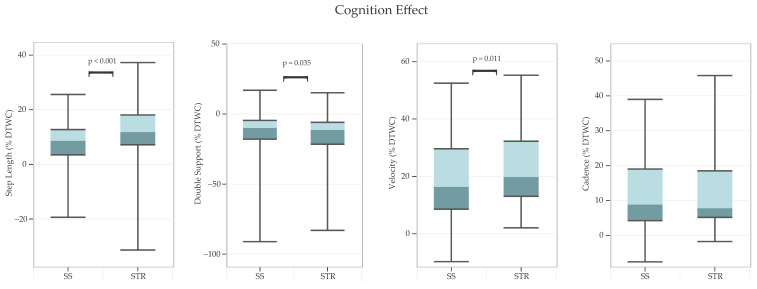
*Cognition effects on dual-task walking cost (DTWC) for various gait parameters*. Note: Boxes represent interquartile ranges, the lighter shade of color represents upper 50% of the data, and error bars represent minimum and maximum values. Positive *y*-axis values indicate greater DTWC, reflecting reductions in step length, velocity, and cadence; whereas double-support DTWC shows the opposite trend, an increase in double-support time. STR = Stroop; SS = serial subtraction.

Regarding main effects, STR resulted in greater gait DTWC than SS on the following gait parameters (Figure 3): step length [F(1, 36) = 32.60, *p* < 0.001, partial η^2^ = 0.48], double support time [F(1, 36) = 4.77, *p* = 0.035, partial η^2^ = 0.12], and velocity [F(1, 36) = 7.21, *p* = 0.011, partial η^2^ = 0.17].

**Figure 4:**
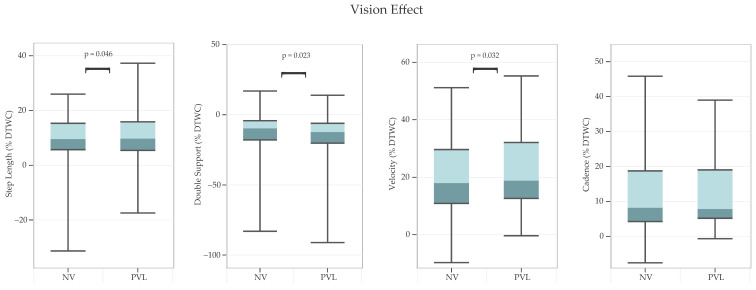
*Vision effects on dual-task walking cost (DTWC) for various gait parameters.* Note: Boxes represent interquartile ranges, the lighter shade of color represents upper 50% of the data, and error bars represent minimum and maximum values. Positive *y*-axis values indicate greater DTWC, reflecting reductions in step length, velocity, and cadence; whereas double-support DTWC shows the opposite trend, an increase in double-support time. NV = normal vision; PVL = peripheral vision loss.

In addition, PVL resulted in greater gait DTWC than normal vision (Figure 4) for: step length [F(1, 36) = 4.27, *p* = 0.046, partial η^2^ = 0.11], double support [F(1, 36) = 5.62, *p* = 0.023, partial η^2^ = 0.14], and velocity [F(1, 36) = 5.00, *p* = 0.032, partial η^2^ = 0.12].

**Figure 5:**
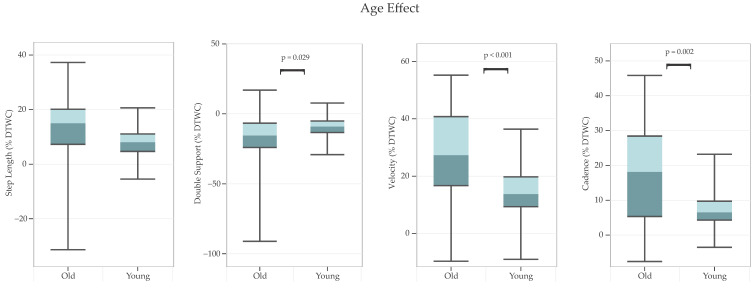
*Main effects of age on dual-task walking cost (%DTWC) for various gait parameters.* Note: Positive *y*-axis values indicate greater DTWC, reflecting reductions in step length, velocity, and cadence; whereas double-support DTWC shows the opposite trend, an increase in double-support time. Boxes represent interquartile ranges, horizontal lines within boxes indicate medians, and error bars represent minimum and maximum values.

Finally, older adults had greater gait DTWC than younger adults (Figure 5) for: double support [F(1, 36) = 5.14, *p* = 0.029, partial η^2^ = 0.13], velocity [F(1, 36) = 13.38, *p* < 0.001, partial η^2^ = 0.27], and cadence [F(1, 36) = 11.38, *p* = 0.002, partial η^2^ = 0.24].

## 4. Discussion

This study investigated the impact of different types of cognitive tasks and vision conditions on gait performance in younger and older adults. Although no significant cognition by vision by age interaction was found, there were significant interactions for step length between cognition and age and between cognition and vision. In terms of main effects, there was greater DTWC across all gait parameters during the inhibitory control task compared to the working memory task. Additionally, greater DTWC was observed under PVL condition compared to normal vision. Finally, older adults exhibited greater DTWC compared to younger adults.

### 4.1. Cognition Effect

The STR task produced greater DTWC in gait performance compared to the SS task. This result is consistent with a previous study that found, among younger adults, the STR task led to greater DTWC in gait velocity compared to a visuo-motor reaction time task, a word list generation task, and a serial subtraction task, and no difference in DTWC among the latter three cognitive tasks [21]. In our study, the greater impact of the STR task extended beyond gait velocity to include step length, and double support time. Because the STR task measures inhibitory control, it requires a considerable amount of attention, information processing, and planning to suppress instinctive responses [46], suggesting that multiple cognitive domains are activated in this task. Past studies have found that brain regions such as the prefrontal cortex, anterior cingulate cortex, insula, middle frontal gyrus, cerebellum, supplementary motor area, and retro-splenial gyrus, are activated by the Stroop task [47]. This is in contrast to tasks of working memory, which are mainly associated with the activation of the prefrontal cortex [48]. This result suggests that tasks requiring inhibitory control elicit greater activation from an extensive network of brain regions compared to working memory tasks, resulting in greater attentional demand when paired with a walking task. The results of our study support and extend previous study [21], indicating that the greater cognitive demand of performing a Stroop task, as opposed to a walking memory task, results in more pronounced DTWC in gait.

### 4.2. Age Effect

Older adults exhibited greater DTWC in gait performance compared to younger adults. Specifically, older adults showed decreased velocity and increased cadence and spent a greater duration in the double support phase while dual tasking. These results are consistent with previous studies with similar designs [2,49,50] and align with the broader literature on age-related changes in gait and fall risk [51,52]. In general, healthy younger adults require minimal attention in gait regulation [12,53], but older adults need higher cognitive control [2,54]. A review study [55] suggests that older adults often have more problems than younger ones in walking and concurrently engaging in another activity due to a general loss of brain mass, a distinctive atrophy of the frontal gray matter, and a white matter hyperintensity [56]. Thus, the changes in gait patterns observed in older adults in our study could indicate the use of greater attention to maintain overall gait stability under DT conditions.

### 4.3. Vision Effect

Peripheral vision loss (PVL) produced greater DTWC for step length, velocity, and double support regardless of age. Our results align with past findings. One study investigated the role of vision in walking and found that peripheral vision was important for establishing and updating accurate representations of spatial structure for navigating [35]. In that study, the obscuration of peripheral vision caused participants to drift off course while walking. Another study emphasized how peripheral visual stimuli drove postural adjustments by influencing body inclination and sway dynamics in healthy subjects while standing [57]. The role of peripheral vision in adaptive locomotion is further underscored by research on visual field occlusion during obstacle negotiation [41]. This study found that, in the PVL condition, gait variability was increased and walking speed was slower than under normal vision condition. This result indicates that while central visual cues (exteroceptive) facilitate feed-forward planning that is necessary for gait adaptations, peripheral visual cues (ex-proprioceptive) are vital for real-time adjustments and provide essential proprioceptive input to fine tune gait [41]. Studies have suggested that, in the PVL condition, “useful field of view” in the brain, which is responsible for processing complex visual stimuli rapidly and accurately, might be affected [58,59].

### 4.4. Cognition X Age Effect

For the cognition and age interaction, DT step length deterioration was greater when an inhibitory control task (e.g., STR) was performed compared to a working memory task (e.g., SS), and this effect was more pronounced in older adults than in younger adults. Past studies found that the Stroop task activates an extensive network of brain regions [47] and the age-related shrinkage of the prefrontal cortex affects higher-level cognitive function [2]. Studies also found that decline in EF among older adults is associated with slower gait speed, increased stride variability, poorer performance on complex mobility tasks, and increased fall risk [11,12,13]. Taken together, these results might explain the greater DTWC in step length among older adults than in younger adults. Our result aligns with a past study that found a similar result such that an auditory Stroop task performed while walking decreased gait speed for older adults but not for younger adults [49]. The different results involving younger adults might be related to the different modality in the Stroop task. Since both cognitive tasks assessed aspects of executive functioning, these findings might also support the frontal lobe theory of cognitive aging, which argues that age-related cognitive changes in older adults are the result of age-related neuroanatomical changes in the frontal lobes, more specifically atrophy in the prefrontal cortex [60].

### 4.5. Cognition X Vision Effect

For the cognition and vision interaction, DT step length deterioration was greater when the inhibitory control task was performed compared to the working memory task, and this effect was more pronounced under PVL condition. Previous studies [61,62,63,64] have consistently highlighted the essential role of visual input in stabilizing gait, especially when cognitive resources are taxed. Marigold and Patla [61] examined the role of visual input, particularly from the lower visual field. Their findings indicate that restricted lower visual input led to increased instability while walking on complex surfaces, which inherently demands higher attentional resources, hinting at the importance of cognitive–visual interaction for stability. Mahoney et al. [64] highlighted that when visual tasks are paired with cognitive tasks, the interaction can place a higher demand on attentional resources, potentially leading to delayed reaction times in people with diminished processing capacity, such as older adults. A recent dual-task study corroborated these findings, illustrating that PVL impacts both gait and cognition, where both young and older adults under visual challenges shifted attention away from cognitive tasks to prioritize walking [38]. Furthermore, research on visual field restrictions [42] reveal that severe peripheral vision loss impairs spatial memory and raises attentional demands during navigation, underscoring the role of peripheral vision in establishing, updating, and navigating spatial representations during walking. These findings align with our results, suggesting that the interaction between cognitive and visual demands critically impacts gait stability.

### 4.6. Limitation

The strengths of this study include investigating the impact of simultaneously performed cognitive tasks under different visual conditions on several gait parameters across different age groups. There is an interdependence of step length, velocity, and cadence, as velocity is inherently influenced by changes in step length and/or cadence. However, our findings demonstrated that the effects of cognition and vision were not the same across the walking costs of these parameters. These results underscore the importance of investigating multiple gait parameters. However, there are limitations in this study. The sample size was small, and the ANOVA analyses tested the main and interaction effects of cognition and vision conditions by comparing their population mean differences and did not provide estimations of parameters. Participants were also required to perform single-tasks before dual-tasks. Although the order of cognitive tasks and vision conditions was counterbalanced to minimize the practice effect, practice might still have influenced cognitive task scores, which we did not analyze. In addition, we instructed participants to hold the Stroop chart at a comfortable reading distance and the holding distance may have slightly varied while walking. However, this variability would likely have occurred across all participants. Finally, the current study focused on the effects of cognition and vision on walking performance, not the effects of walking and vision on cognitive performance. Studying cognitive cost under dual-tasking might be able to provide more information, such as the strategies (e.g., prioritization) that participants employed while dual-tasking and the impact of walking on cognitive performance.

## 5. Conclusions

The current study examined multiple gait variables as outcome measures, providing insight into the role of subsets of executive function—inhibition and working memory—and the impact of PVL on gait performance in younger and older adults under a dual-task (DT) paradigm. Greater DTWC in gait performance was observed in the STR task compared to the SS task, in PVL compared to normal vision, and in older adults compared to younger adults. Future research could investigate complex relationships among various cognitive tasks, multiple levels of walking difficulties, and different degrees of visual impairment across different age groups.

## Figures and Tables

**Table 1:** Mean (M) and standard deviation (SD) of various gait parameters for younger and older adults during single task walking and dual task walking. The dual tasks involved walking performing either (a) serial subtraction by 3s (SS) or (b) the Stroop task (STR). Both single task and dual task walking were performed under normal vision (NV) and peripheral vision loss conditions (PVL).

			Step Length		Double Support Time		Velocity		Cadence	
			cm.	%DTWC	%	%DTWC	cm./sec.	%DTWC	step/min.	%DTWC
Younger (n = 20)	NV	Walk Only	72.1 (5.4)		24.0 (4.4)		135.7 (16.7)		113.6 (8.9)	
		Walk + STR	65.9 (7.1)	8.8 (4.9)	26.1 (4.7)	−8.7 (8.3)	115.5 (21.2)	15.1 (8.8)	106.0 (10.2)	6.7 (5.5)
		Walk + SS	66.9 (6.7)	7.3 (6.1)	26.1 (4.8)	−8.7 (8.0)	118.5 (22.8)	14.5 (7.3)	106.2 (11.1)	6.5 (6.7)
	PV	Walk Only	70.8 (6.4)		24.1 (4.7)		133.8 (20.4)		113.6 (9.1)	
		Walk + STR	63.8 (6.5)	9.9 (4.4)	26.7 (4.8)	−11.3 (6.7)	110.9 (20.9)	17.3 (7.3)	104.9 (10.8)	7.8 (4.4)
		Walk + SS	66.1 (6.7)	6.6 (4.4)	26.3 (4.5)	−9.6 (5.0)	114.9 (23.1)	17.3 (7.3)	104.4 (11.6)	8.2 (4.9)
Older(n = 18)	NV	Walk Only	66.9 (12.3)		27.2 (4.3)		125.3 (33.2)		11.9 (14.8)	
		Walk + STR	57.7 (12.2)	13.4 (12.9)	32.0 (6.9)	−18.1 (19.4)	88.4 (18.9)	27.6 (12.1)	93.6 (14.3)	15.5 (12.9)
		Walk + SS	60.3 (11.7)	9.5 (10.2)	30.5 (4.9)	−12.7 (13.5)	92.0 (26.8)	25.5 (16.2)	91.5 (15.2)	17.6 (13.0)
	PV	Walk Only	66.8 (12.6)		26.9 (4.7)		125.6 (32.9)		112.7 (15.1)	
		Walk + STR	54.4 (11.9)	18.4 (9.5)	32.3 (5.1)	−20.7 (13.3)	84.0 (24.7)	32.0 (14.4)	93.5 (13.2)	16.3 (11.2)
		Walk + SS	59.4 (11.4)	10.5 (11.2)	31.8 (7.6)	−18.8 (22.1)	89.7 (25.6)	26.7 (17.7)	90.6 (16.6)	18.9 (14.4)

## Data Availability

Data are contained within the article.
